# Nursing team’s perceptions about care for pregnant women in a psychiatric unit

**DOI:** 10.1590/0034-7167-2023-0186

**Published:** 2024-12-16

**Authors:** Helena Moro Stochero, Suzinara Beatriz Soares de Lima, Juliana Silveira Colomé, Dirce Stein Backes, Mara Regina Caino Teixeira Marchiori

**Affiliations:** I Universidade Franciscana. Santa Maria, Rio Grande do Sul, Brazil; II Universidade Federal de Santa Maria. Santa Maria, Rio Grande do Sul, Brazil

**Keywords:** Mental Health Assistance, Pregnancy, Hospitalization, Nursing Care, Obstetric Nursing., Asistencia de Salud Mental, Embarazo, Hospitalización, Atención de Enfermería, Enfermería Obstétrica.

## Abstract

**Objectives:**

to understand the nursing team’s perception in relation to the care provided to pregnant women with mental disorders admitted to a psychiatric hospital unit.

**Methods:**

Convergent Care Research carried out between August and December 2021, through semi-structured interviews with 25 nursing professionals from a Psychiatric Unit from a reference Hospital in Southern Brazil.

**Results:**

the organized and analyzed data resulted in two thematic categories: Technical, generic and impersonal care; and From impersonality to the singularity of nursing care. Ensuring unique care for pregnant women with mental disorders means giving them a meaning of existence and providing care from a multidimensional and continuous perspective.

**Final Considerations:**

nursing care for pregnant women in psychiatric hospitalization requires continuous professional qualification, interactive technologies and support for the nursing process, in addition to promoting singular and multidimensional care.

## RESUMEN

**Objetivos:**

conocer la percepción del equipo de enfermería sobre los cuidados prestados a las mujeres embarazadas con trastornos mentales ingresadas en una unidad de hospitalización psiquiátrica.

**Métodos:**

se trata de una Investigación Convergente Asistencial realizada entre agosto y diciembre de 2021 mediante entrevista semiestructurada entre 25 profesionales de enfermería de una Unidad Psiquiátrica de un Hospital referencia del Sur de Brasil.

**Resultados:**

los datos organizados y analizados dieron lugar a dos categorías temáticas: Cuidados técnicos, genéricos e impersonales; y De la impersonalidad a la singularidad en los cuidados de enfermería. Garantizar un cuidado singular y continuo, desde una perspectiva multidimensional, le brinda un sentido a la existencia de las gestantes con trastornos mentales.

**Consideraciones Finales:**

los cuidados de enfermería para mujeres embarazadas en hospitalización psiquiátrica requieren una cualificación profesional continua, tecnologías interactivas y apoyo al proceso de enfermería, así como la promoción de cuidados singulares y multidimensionales.

## INTRODUCTION

Pregnancy is an important milestone in a woman’s life, permeated by a journey of uncertainties related to the development of the fetus, type of birth, breastfeeding and other lessons learned. Furthermore, pregnant women experience a unique period, as they need to adjust to the different roles of being a mother and caregiver, which can adversely affect their mental health^(1)^.

Studies have shown that psychological dysfunctions can occur during pregnancy, including anxiety, depression and stress. Perinatal anxiety is present in approximately 20.7% of pregnant women, depression in approximately 11.9% of women and perinatal stress in approximately 84% of women^(2-4)^. In addition to the potential impact on the health of women and children, irreversible mental disorders may occur and, sometimes, hospitalizations of pregnant women in psychiatric hospitals may be necessary.

The nursing care provided to pregnant women treated in emergencies or in psychiatric hospital units due to mental disorders has, however, been treated with a biological focus, in which the physical component is dissociated from the mental and psychological dimension of the woman. Studies have shown that this dissociation constitutes a gap in the implementation of the Nursing Process, and that some circumstances inherent to nursing care do not allow its implementation as a singular and multidimensional process^(5-7)^.

A study that addressed the perception of nurses regarding the development of nursing practice in long-stay psychiatric inpatient units demonstrated that professional practice in these units and the organization of care are generally focused on fragmented and dichotomous tasks, which mostly result in bureaucratic and automated interventions^(8)^. Along this path, care is reduced to a mere act of doing, that is, unaccompanied by critical-reflective reasoning to plan and intervene in order to consider the psychic and emotional specificities of pregnant women.

To overcome the technical and routine focus of care in psychiatric units, it is necessary to expand the multidimensional understanding of pregnant women and the necessary approaches according to the needs of each one of them. In this journey, psychological support is essential for women to prepare for the experience of hospitalization and adapt to the necessary changes^(9)^. Furthermore, it is essential to promote care environments that are safe and favorable to the promotion of comprehensive care in psychiatric units, so that pregnant women with mental disorders feel welcomed and respected in their uniqueness^(10)^.

Studies justify this technical care practice by demonstrating the difficulties related to work overload, dynamics and turnover, as well as the unpreparedness of professionals to deal with and act on emotional adversities, especially in pregnant women. Furthermore, they highlight the validity of the hegemonic biomedical model in hospital institutions, the nature of the assistance provided in psychiatric units, the lack of appropriate technologies for the systematization of care^(11-13)^, among other justifications.

Although pregnancy, in itself, does not characterize a risk factor for the emergence or recurrence of mental adversities that require hospitalization in psychiatric units, studies^(14,15)^ show high rates of mental disorders among this specific population. Despite its high prevalence in pregnant women, there is a lack of studies, and mainly of strategies that lead to better practices in psychiatric inpatient units.

Given the adversities and the way in which mental health care for pregnant women with mental disorders and in situations of psychiatric hospitalization have been highlighted, the need to expand studies is emphasized to understand how care is being promoted by nursing professionals and which difficulties surround the work and care process for pregnant women hospitalized in a psychiatric hospital inpatient unit. From this perspective, the question is: What is the nursing team’s perception of the care provided to pregnant women with mental disorders admitted to a psychiatric hospital unit?

## OBJECTIVES

To understand the nursing team’s perception of the care provided to pregnant women with mental disorders admitted to a psychiatric hospital unit.

## METHODS

### Ethical aspects

This study followed the guidelines established in Resolution of the National Health Council No. 466/2012 and the recommendations of Circular Letter No. 2/2021, associated with online research^(16)^. The research project was approved by the Research Ethics Committee. All participants signed the Free and Informed Consent Form. The anonymity of the participants was guaranteed by the alphanumeric coding of the interviews, in which the statements were identified, N1, N2... (Nurse), T1, T2... (Nursing Technician) and A1, A2... (Nursing Assistant).

### Theoretical-methodological framework

The assumptions of the Holistic Theory were used as a theoretical framework and qualitative research as a methodological framework. In this journey, the human being is considered as a multidimensional module, who needs understanding and care for their physiological needs (physiological aspects), minds (emotional aspects) and souls (spiritual aspects)^(17)^.

### Type of study and methodological procedures

This is a Convergent Care Research (CCR), chosen with the purpose of prospecting improvements that can be implemented in the context of nursing care, in addition to enabling solutions to emerging problems^(18)^. The investigation process was conducted based on the four stages proposed by the CCR, namely: Conception, Instrumentation, Inspection and Analysis^(18)^. Throughout the construction process, the Consolidated Criteria for Reporting Qualitative Research (COREQ) criteria were considered.^(19)^.

### Study setting

The research was carried out in a Psychiatric Hospital Inpatient Unit, within a reference public institution in southern Brazil. This scenario was intentionally selected because it is a highly complex health service and a reference in monitoring pregnant women with mental disorders. This Unit has 30 beds distributed in male and female wards. The nursing team is composed by 13 nurses, including a coordinator, 16 nursing technicians and 6 nursing assistants.

### Study participants

Among the team of 35 nursing professionals, 25 professionals agreed to participate in this study, including seven nurses, sixteen nursing technicians and two nursing assistants who work in the aforementioned Psychiatric Unit. The inclusion criterion was to join the nursing team from the same unit who also had been working there for at least six months, considering that this period allows the professional to understand how the work process occurs. And as an exclusion criterion: being on leave from work during the data collection period. Based on these criteria, only three team professionals were excluded.

### Data collection and organization

Data were collected between August and December 2021, through semi-structured interviews. The data collection instrument included aspects related to the personal and professional characteristics of the participants, such as: training, age, time since training, time working in the hospital and in the unit, in addition to guiding questions, namely: How do you perceive your assistance to pregnant women with mental disorders admitted to this Unit? What do you consider important to say about the way in which care for pregnant women has been developed in the Psychiatric Hospital Inpatient Unit?

Due to the pandemic period, which restricted researchers’ access to health services, the interviews were carried out, after prior contact with the participants, synchronously through digital platforms suggested by the participants. The days and times stipulated by the participants for carrying out the interviews were also considered. Attention was paid to online data collection strategies in qualitative research in the health area, previously listed in this study^(20)^.

### Data analysis

The data were analyzed based on the CCR guiding assumptions^(18)^. Thus, the analysis was conducted based on the following steps: apprehension, synthesis, theorization and transfer. Data collection was conducted by reading the material and analyzing the reports in the order in which they were obtained. The synthesis of the central ideas of the interviews was carried out based on the reordering of empirical data according to their characteristics and relationships between them until reaching the final analysis. In the theorization stage, the data were articulated with the theoretical framework. Finally, the results were transferred and replicated to the participants involved in the research process.

The three dimensions proposed by the CCR method were considered in the analysis process: Dimension I - Dimension of the sociodemographic profile of professionals; Dimension II - Professional dimension; Dimension III - Dimension of autonomy.

## RESULTS

In relation to Dimension I, the largest number of participants were aged between 40 and 65 years old. Regarding professional training time, the variation between nurses was 14 and 35 years, between technicians between 3 and 36 years and between nursing assistants between 24 and 40 years. Among the nurses, one had a doctorate and eight had a master’s degree. Among the technicians and assistants, 11 professionals had a higher education degree and two had a master’s degree. And, in relation to the length of professional experience in the aforementioned psychiatric unit, there was a minimum variation of six months and a maximum of 38 years.

Dimensions II and III related to the professional and autonomy were integrated and the organized data were analyzed and resulted in two thematic categories, namely: Technical, generic and impersonal care; and From impersonality to the singularity of nursing care.

### Technical, generic and impersonal care

The care provided to pregnant women admitted to the psychiatric inpatient unit is no different from the care provided to other hospitalized patients. In some cases, the pregnant woman is considered in her nature, but the care ends up being generic, superficial and impersonal. This way of proceeding and caring may be related to inexperience and/or inability to deal with pregnant women hospitalized with mental disorders.

*Individual care for pregnant women is incomplete and, at times, insufficient and superficial.* (T1)

*The service ends up being more technical with general care and provides little attention to the development of more specific care for pregnant women’s health.* (N2)

*The patient is monitored in a generic way, like all other patients.* (T5)

*Care becomes deficient because we are not prepared to treat patients in these conditions.* (T14)

Participants also emphasized the excessive standards, protocols and daily routines that must be followed in the Unit and that end up mechanizing and depersonalizing the work and care process of nursing professionals. The thoughtless and automated routine reduces care to a mere technical, punctual and routine task, unaccompanied by critical-reflective reflection and without taking into account the unique and individualized care of pregnant women, as expressed below:

*Here at the unit I provide non-specific care due to the routines established in this sector. Treatment is not individualized. Everything seems to be mechanical, technical.* (N4)

It was noted, in other statements, that the nursing team is not properly qualified to care for pregnant women hospitalized in a psychiatric hospital inpatient unit, although they recognize that the pregnant woman goes through several physiological and psychological changes and that she needs to be welcomed and supported, not as ‘another patient’ (number), but as someone who experiences a unique moment in her life and in the life of her child. Therefore, this unique care for pregnant women is sometimes trivialized to the detriment of rigid rules, protocols and thoughtless routines. Sometimes, the pregnant woman is recognized, but only when she presents some complications and requires some differentiated care.

*Care for pregnant women in the service ends up unnoticed if they do not present any complications.* (N2)

*I believe that, like me, the team is not sufficiently trained.* (N4)

*Lack of preparation for this audience.* (N7)

*Difficulty with the team’s lack of preparation for specific care for pregnant women.* (N1)

*I notice the lack of training.* (A1)

It was also noted in the statements of some participants the difficulties related to the correct and complete recording of information in the pregnant women’s medical records in relation to the specificities of the mother-fetus binomial. This process is sometimes reduced to a secondary action, dissociated from integrative care, as expressed:

[...] *records need to be improved. Getting more details about the mother and baby.* (A2)

*Registration is not assumed as care to be provided for mother and baby* [...]. (T7)

*In the nursing process as an instrument to outline care, there are no significant changes between the pregnant woman and a common patient.* (N6)

*It is necessary to include specific care for pregnancy in nursing prescriptions, which provide information about the pregnant woman and her baby* [...]. (N4)

Hospitalization often depersonalizes the person and reduces them to a mere patient, suffering from a disease and in need of treatment. Therefore, it is perceived and treated only as a “sick person” and reduced to the physical dimension, without considering the human multidimensionality of care. In this logic, all other equally relevant dimensions are relegated to a secondary level. Under this approach, one participant mentioned that care must be expanded and the act of caring needs to include, in addition to the biological view, the person/patient in their entirety and multidimensionality. How, however, can we expand this perception of nursing/health care?

### From impersonality to the singularity of nursing care

Although participants reported little knowledge and ability to deal with and care for pregnant women, on the other hand, there was a desire for pregnant women to be welcomed, supported and cared for in their uniqueness and beyond their mental illness, according to statements:

*I realize that pregnant women cannot be treated as just another patient. She is at a special moment in her life and her son’s life.* (TN3)

*I consider it a complex work, which requires greater theoretical-practical foundation to assist in the management and care of pregnant women, as it is a special moment in their lives.* (AI)

Pregnancy, in the understanding of the participants, involves an integrative dynamic that involves the mother-baby binomial. From this perspective, care for pregnant women also needs to understand and embrace their unborn child. Technological tools and/or specific operational procedures that support care in psychiatric units were highlighted as strategies to qualify the work process.

*There is no protocol of specific routines and guidelines for caring for pregnant women. We would have to think something in this direction.* (T1)

*I miss a specific resource for pregnant women. They need to be treated in their individuality. These resources would help us individualize the care process.* (N5)

In addition to operational procedures aimed at pregnant women, other participants mentioned the need for technologies that ensure multidimensional nursing care, from planning to execution. In this sense, they recognize that nursing care demands specific knowledge for decision-making, based on critical-reflective reasoning.

*As improvements, there should be protocols to guide pregnant women’s routines. These would help us in decision making.* (T1)

*I suggest that some technology be proposed and established to support the implementation of specialized care for hospitalized pregnant women.* (N4)

*There is a lack of planning and systematization for decision making.* (N7)

The difficulties related to making a decision about the best behavior to be planned and adopted with the pregnant woman were highlighted in several statements. In general, nursing professionals expressed interest in expanding their perception and recognized the need for specific knowledge to deal with pregnant women hospitalized with mental disorders.

*I believe that the biggest difficulty is related to the approaches, how to monitor this pregnant woman in the service, knowing what specific care to use in the assistance. We need qualifications.* (N2)

It was noticed, in several statements, that nursing professionals feel unprepared to deal with the psychiatric specificities of hospitalized pregnant women. This thinking demonstrates that the Nursing Process needs to be adapted to different realities and that professionals, equally, need to be equipped based on the specificities of each context and existential reality.

*I think that assistance is limited, because pregnant women have more restrictions and we don’t know how to deal with.* (T14)

*Lack of practice with this type of audience.* (T12)

One of the fundamental tasks of the human condition is the act of caring, which is consolidated through interactions, associations, technologies and lifelong learning. From this perspective, care is embedded in the existential dynamics of each and every human being, who must be welcomed as unique in their different stages of life. Thus, ensuring unique nursing care for pregnant women with mental disorders means giving them meaning in life, existence, integral health and contributing to the healthy development of their child during pregnancy.

## DISCUSSION

Psychiatric hospital units are generally recognized for their control over daily routines and rigid protocol determinations, which weaken interactions and manifestations of individuality. In these units, patients are, most of the time, treated in a generic and impersonal way and generally reduced to mental patients^(21)^. This thinking and acting has been questioned, more specifically following the Psychiatric Reform, which provides for a comprehensive and humanized look at mental health^(22)^.

Although the quality and effectiveness of mental health care have reached new heights in recent decades, therapeutic revolutions in psychiatry still continue to (re)produce stigmas. A study demonstrates that the demerit related to mental patients and the consequent fragmentation of care in this area constitute a barrier to treatment and recovery. Furthermore, the stigma associated with the disease - mental patient has impacted the search behavior and healthy living of these users^(23)^.

Mental disorders in pregnant women are even more complex. In addition to the stigma of mental illness, pregnant women face the disadvantage of lack of specific access to perinatal mental health services, the weak qualification of health professionals in this specific area, the lack of integrative and collaborative approaches based on scientific evidence, among others. factors. A study shows that the stigma of mental disorders is largely a barrier to seeking support in health services and, consequently, the topic remains unexplored in most low and medium development countries^(24)^.

In another study, it was noted that pregnant women with pre-existing mental illnesses present an increased risk of obstetric and neonatal outcomes compared to pregnant women without previous mental disorders^(25)^. Previous mental illness in pregnant women increases obstetric risks and, therefore, demands unique and comprehensive assistance, considering that poor quality of care can influence the mother-child bond and incur medium and long-term implications for both mother and child. in the child, partners/families^(26)^.

In the same direction, it was evident that pregnant women hospitalized in psychiatric units are considered users with a greater number of acute illnesses compared to those who are treated and monitored in outpatient clinics^(27,28)^. There are no investigations, however, that demonstrate how the severity of mental disorders in pregnant women, according to the quality of care received, may be associated with adverse obstetric outcomes.

Considering the prevalence and impact of mental disorders during pregnancy and, at the same time, a less unique and integrated care process, new studies are essential in order to clarify the real impacts and promote advances related to intervention approaches in mental health. The results of this research demonstrated that there is a lack of professional preparation in relation to the care of pregnant women hospitalized in psychiatric units, although there is a desire for qualification in the area, in order to contemplate and ensure the individuality of each user (pregnant woman).

In addition to the place of care for pregnant women with mental disorders, the results of the present study intuited points of discussion related to intervention approaches by health professionals and which relate to the understanding of care. Health care is, par excellence, a complex phenomenon due to its unique, original and transformative character. Driven through multiple relationships, interactions and systemic associations, care always has as its ultimate purpose the well-being of the person/user at an individual and collective level, regardless of their emotional state or dimension affected^(29)^.

In this expanded direction, the uniqueness of nursing care has a direct relationship with the meaning of work, ambience, reception, empathy and respect for differences, regardless of the nature of the disease or the emotional state of the patient/user. The nurse has a relevant and recognized role in prospectively leading the care process, based on horizontal, dialogical and collegiate technologies^(29)^. To this end, it is necessary to expand perception and knowledge about the real meaning of health care, which must transcend the fragmentary and technical logic - mental illness and achieve human integrality and multidimensionality.

Nursing care consists, in this evolutionary dynamic, of making transpersonal and multidisciplinary efforts towards others, in this case pregnant women with mental disorders, with the aim of protecting, promoting and welcoming them in their uniqueness, regardless of their state, situation or commitment^(30,31)^. This thinking implies going beyond the biological perception of health care and, prospectively, achieving an integrative thinking in which the caregiver/professional perceives themselves in care and as care.

A study^(32)^ demonstrates that, although it is a complex concept, expanded and multidimensional care captures the human person in their uniqueness. Under this approach, care provides a deep understanding of each patient/user, based on their specific needs and demands. In this sense, in addition to contributing to user satisfaction, it results in individual and collective well-being.

The quality and impact of nursing care are determined, in short, by the quality of relationships and interactions and respect for human singularities. Study^(29)^ reinforces this thinking, considering that care is determined by respectful and unique reception to the needs of each user, family, community, regardless of the situation in which they find themselves.

### Study limitations

The limitations of this study are associated with the non-participation of all nursing team professionals invited for this purpose, considering that the results could have been much more developed and solid. This non-participation may be related to the overload of activities due to the pandemic period. Another limitation may be associated with the fact that this study was carried out in a single psychiatric unit, which makes generalizations unfeasible.

### Contributions to the field of Nursing

The study will contribute to the organization of nursing care for pregnant women hospitalized in psychiatric hospital units, based on singular and multidimensional approaches. In this way, the results of this study may leverage new management strategies, within the scope of the Federal Nursing Council (COFEN), to understand the Nursing Process in these specific units, often lacking specific studies, qualifications and technologies.

## FINAL CONSIDERATIONS

The organization of nursing care for pregnant women hospitalized in a psychiatric hospital unit, in the perception of the nursing team, translates into a movement that involves techniques, routines and protocols. Nursing care for pregnant women in psychiatric hospitalization requires continuous professional qualification, interactive technologies and support for the nursing process, in addition to promoting singular and multidimensional care.

Ensuring unique nursing care for pregnant women, especially those hospitalized in psychiatric units, means giving them meaning in life, existence and conducting care, from a multidimensional and multidisciplinary perspective, in order to ensure completeness and continuity of health care. It means rethinking approaches and conducting care from these perspectives. In short, it consists of thinking about care in a collaborative and interconnected way with other levels of complexity, in order to ensure the integrality and continuity of health care.
