# Supplementary material for: Nursing team’s perceptions about care for pregnant women in a psychiatric unit
Source: Rev Bras Enferm. 2024 Dec 16;77(6):e20230186. doi: 10.1590/0034-7167-2023-0186 (PMC11654521; doi:10.1590/0034-7167-2023-0186)
Supplement: Translation

# Translation: Percepções da equipe de enfermagem sobre o cuidado às gestantes em unidade psiquiátrica

**DOI:** 10.1590/0034-7167-2023-0186pt

**Authors:** Helena Moro Stochero, Suzinara Beatriz Soares de Lima, Juliana Silveira Colomé, Dirce Stein Backes, Mara Regina Caino Teixeira Marchiori

**Affiliations:** I Universidade Franciscana. Santa Maria, Rio Grande do Sul, Brasil; II Universidade Federal de Santa Maria. Santa Maria, Rio Grande do Sul, Brasil

**Keywords:** Assistência à, Saúde Mental, Gravidez, Hospitalização, Cuidados de Enfermagem, Enfermagem Obstétrica.

## Abstract

**Objetivos:**

conhecer a percepção da equipe de enfermagem sobre cuidado prestado às gestantes com transtornos mentais internadas em uma unidade de internação hospitalar psiquiátrica.

**Métodos:**

Pesquisa Convergente Assistencial realizada entre agosto e dezembro de 2021, por meio de entrevista semiestruturada com 25 profissionais de enfermagem de uma Unidade Psiquiátrica de um Hospital de referência do Sul do Brasil.

**Resultado:**

os dados organizados e analisados resultaram em duas categorias temáticas: Cuidado tecnicista, genérico e impessoal; e Da impessoalidade à singularidade do cuidado em enfermagem. Assegurar um cuidado singular às gestantes com transtornos mentais significa atribuir-lhes significado de existência e conduzir o cuidado, a partir de uma perspectiva multidimensional e continuada.

**Considerações Finais:**

o cuidado de enfermagem às gestantes, em situação de internação psiquiátrica, demanda contínua qualificação profissional, tecnologias interativas e de apoio ao processo de enfermagem, além da promoção de um cuidado singular e multidimensional.

## INTRODUÇÃO

A gestação é um marco importante na vida de uma mulher, permeado por um percurso de incertezas relacionadas ao desenvolvimento do feto, tipo de parto, amamentação e outros aprendizados. Além disso, a gestante vivencia um período singular, visto que a mesma necessita ajustar-se aos diferentes papéis de ser mãe e cuidadora, o que pode afetar adversamente a sua saúde mental^(1)^.

Estudos demonstram que podem ocorrer disfunções psicológicas no período gestacional, dentre as quais destacam-se a ansiedade, a depressão e o estresse. A ansiedade perinatal está presente em cerca de 20,7% das gestantes, a depressão em aproximadamente 11,9% das mulheres e o estresse perinatal em cerca de 84% das mulheres^(2-4)^. Além de potencial impacto na saúde das mulheres e crianças, podem ocorrer transtornos mentais irreversíveis e, por vezes, resultarem na internação de gestantes em unidades hospitalares psiquiátricas.

O cuidado de enfermagem prestado às gestantes atendidas em emergências ou em unidades hospitalares psiquiátricas em função de transtornos mentais tem sido tratado, no entanto, com enfoque biologista, em que se dissocia a parte física da dimensão mental e psicológica. Estudos evidenciam que essa dissociação se constitui em uma limitação à efetivação do Processo de Enfermagem em sua concepção singular e multidimensional^(5-7)^.

Estudo que abordou a percepção de enfermeiros quanto ao desenvolvimento da prática de enfermagem em unidades de internação psiquiátricas de longa permanência demonstrou que a prática profissional nestas unidades e a organização do cuidado, geralmente, se centra em tarefas fragmentadas e dicotômicas as quais resultam, na maioria das vezes, em intervenções burocráticas e automatizadas^(8)^. Nesse percurso, o cuidado reduz-se a um mero fazer, isto é, desacompanhado de raciocínio crítico-reflexivo para planejar e intervir de modo a considerar às especificidades psíquicas e emocionais das gestantes.

Para superar o enfoque técnico-biologista da assistência em unidades psiquiátricas é preciso ampliar a compreensão e abarcar as múltiplas dimensões das gestantes, de acordo com as necessidades de cada uma delas. O apoio psicológico é, nesse percurso, de fundamental importância para que a mulher elabore a experiência da hospitalização e se adapte às mudanças necessárias^(9)^. Além disso, é essencial que se promova ambientes de cuidado que sejam seguros e favoráveis à dinamização do cuidado integral em unidades psiquiátricas, de modo que a gestante com transtorno mental se sinta acolhida e respeitada em sua singularidade^(10)^.

Estudos justificam essa prática tecnicista de cuidado, ao demostrarem as dificuldades relacionadas à sobrecarga e rotatividade no trabalho, bem como ao despreparo dos profissionais para lidarem e atuarem com as adversidades emocionais, especialmente em gestantes. Destacam, ainda, a supremacia do modelo biomédico nas instituições hospitalares, a natureza da assistência prestada nas unidades psiquiátricas, a falta de tecnologias apropriadas à sistematização do cuidado^(11-13)^, dentre outras justificativas.

Embora a gestação, por si só, não caracterize um fator de risco para o surgimento ou recorrência de adversidades mentais que demandam internações em unidades psiquiátricas, estudos^(14,15)^ evidenciam elevadas taxas de transtornos mentais entre essa população específica. Apesar de sua alta prevalência em gestantes, percebe-se a carência de estudos e, principalmente, de estratégias que conduzam às melhores práticas nas unidades de internação psiquiátrica.

Dadas as adversidades e o modo como a assistência à saúde mental de gestantes com transtornos mentais e em situação de internação psiquiátrica vem sendo evidenciada, enfatiza-se a necessidade de ampliar as investigações para compreender como o cuidado vem sendo promovido pelos profissionais de enfermagem e quais as dificuldades que circundam o processo de trabalho e de cuidado às gestantes internadas em uma unidade de internação hospitalar psiquiátrica. Sob esse enfoque, questiona-se: Qual a percepção da equipe de enfermagem sobre o cuidado prestado às gestantes com transtornos mentais internadas em uma unidade de internação hospitalar psiquiátrica?

## OBJETIVOS

Conhecer a percepção da equipe de enfermagem sobre cuidado prestado às gestantes com transtornos mentais internadas em uma unidade de internação hospitalar psiquiátrica.

## MÉTODOS

### Aspectos éticos

O estudo seguiu as diretrizes estabelecidas pela Resolução do Conselho Nacional de Saúde n° 466/2012 e as recomendações do Ofício Circular nº 2/2021, associadas às investigações na modalidade online^(16)^. O projeto de pesquisa foi aprovado pelo Comitê de Ética e Pesquisa e todos os participantes assinaram o Termo de Consentimento Livre e Esclarecido. O anonimato dos participantes foi garantido pela codificação alfanumérica das entrevistas, no qual as falas foram identificadas, E1, E2... (Enfermeiro), T1, T2... (Técnico de Enfermagem) e A1, A2... (Auxiliar de Enfermagem).

### Referencial teórico-metodológico

Utilizou-se como referencial teórico os pressupostos da Teoria Holística e como referencial metodológico a pesquisa qualitativa. Considera-se, nesse percurso, o ser humano como unidade multidimensional, que necessita ser apreendido a partir de suas necessidades fisiológicas, mentais, sociais e espirituais^(17)^.

### Tipo de estudo e procedimentos metodológicos

Trata-se de uma Pesquisa Convergente Assistencial (PCA), elegida com o propósito de prospectar melhorias no contexto do cuidado de enfermagem, além de possibilitar soluções para problemas emergentes^(18)^. O processo de investigação foi conduzido com base nas quatro etapas propostas pela PCA, quais sejam: Concepção, Instrumentação, Perscrutação e Análise^(18)^. Considerou-se, no o processo de construção, os critérios do *Consolidated Criteria for Reporting Qualitative Research* (COREQ)^(19)^.

### Cenário do estudo

A pesquisa foi desenvolvida em uma Unidade de Internação Hospitalar Psiquiátrica, instituição pública de referência no sul do Brasil. Esse cenário foi selecionado, intencionalmente, por ser o campo de atuação de uma das pesquisadoras e por ser um serviço de referência no acompanhamento de gestantes com transtornos mentais. A referida Unidade dispõe de 30 leitos distribuídos em enfermarias masculinas e femininas. A equipe de enfermagem é composta por 13 enfermeiros, sendo um coordenador, 16 técnicos de enfermagem e seis auxiliares de enfermagem.

### Participantes do estudo

Dentre a equipe de 35 profissionais de enfermagem, participaram deste estudo 25 profissionais, dos quais sete enfermeiros, dezesseis técnicos de enfermagem e dois auxiliares de enfermagem que atuam na referida Unidade Psiquiátrica. O critério de inclusão foi integrar a equipe de enfermagem da referida unidade e ter trabalhado, no mínimo, seis meses nesta unidade, tendo em vista que esse período permite ao profissional compreender o modo como ocorre o processo de trabalho. E como critério de exclusão, estar afastado do serviço no período de coleta de dados por algum motivo justificado. Com base nestes critérios, apenas três profissionais da equipe foram excluídos.

### Coleta e organização dos dados

Os dados foram coletados entre agosto e dezembro de 2021, por meio de entrevista semiestruturada. O instrumento de coleta de dados incluiu aspectos relacionados à caracterização pessoal e profissionais dos participantes, tais como: formação, idade, tempo de formação, tempo de atuação no hospital e na unidade, além de questões norteadoras, quais sejam: Como você percebe a sua assistência às gestantes com transtornos mentais internadas nessa Unidade? O que você considera importante falar sobre o modo como o cuidado às gestantes vem sendo desenvolvido na Unidade de Internação Hospitalar Psiquiátrica?

Em decorrência do período pandêmico, que restringiu o acesso de pesquisadores nos serviços de saúde, as entrevistas foram realizadas, após contato prévio com os participantes, de modo síncrono por meio de plataformas digitais sugeridas pelos participantes. Considerou-se, ainda, os dias e horários estipulados pelos participantes para a realização das entrevistas. Atentou-se para as estratégias de coleta de dados online nas pesquisas qualitativas da área da saúde, previamente elencadas nesse estudo^(20)^.

### Análise dos dados

Os dados foram analisados com base nos pressupostos orientadores da PCA^(18)^. Assim, a análise foi conduzida a partir das seguintes etapas: apreensão, síntese, teorização e transferência. A apreensão dos dados foi conduzida mediante a leitura do material e a análise dos relatos na ordem em que foram obtidos. A síntese das ideias centrais das entrevistas foi realizada com base na reordenação dos dados empíricos de acordo com as suas características e relações entre si, até alcançar a análise final. Na etapa de teorização, os dados foram articulados com o referencial teórico. Conduziu-se, por fim, a transferência e a replicação dos resultados para os participantes envolvidos no processo de pesquisa.

Considerou-se no processo de análise as três dimensões propostas pelo método PCA: Dimensão I - Dimensão do perfil sociodemográfico dos profissionais; Dimensão II - Dimensão profissional; Dimensão III - Dimensão da autonomia.

## RESULTADOS

Em relação a Dimensão I, o maior número de participantes possuía idade entre 40 e 65 anos. Quanto ao tempo de formação profissional, a variação entre os enfermeiros foi de 14 e 35 anos, entre os técnicos de 03 e 36 anos e entre os auxiliares de enfermagem de 24 e 40 anos. Dentre os enfermeiros, um possuía doutorado e oito possuíam mestrado e os técnicos e auxiliares, 11 profissionais possuíam curso superior e dois possuíam mestrado. E, em relação ao tempo de atuação profissional na referida unidade psiquiátrica, obteve-se uma variação mínima de seis meses e máxima de 38 anos.

As dimensões II e III relacionadas ao profissional e à autonomia foram integralizadas e os dados organizados foram analisados e resultaram em duas categorias temáticas, quais sejam: Cuidado tecnicista, genérico e impessoal; e Da impessoalidade à singularidade do cuidado de enfermagem.

### Cuidado tecnicista, genérico e impessoal

O cuidado às gestantes internadas na unidade de internação psiquiátrica não se difere do cuidado dispensado aos demais pacientes internados. Em alguns casos a gestante é considerada em sua natureza, mas o cuidado acaba por ser genérico, superficial e impessoal. Esse modo de proceder e cuidar pode estar relacionado à inexperiência e/ou à incapacidade de lidar com as gestantes internadas com transtornos mentais.

*O cuidado individual a gestante é incompleto e, por vezes, insuficiente e superficial.* (T1)

*O serviço acaba por ser mais tecnicista com cuidados gerais e dá pouca atenção ao desenvolvimento de um cuidado mais específico à saúde da gestante.* (E2)

*A paciente é acompanhada de maneira genérica, como todos os outros pacientes.* (T5)

*O cuidado se torna deficiente, devido não estarmos preparados para tratar de pacientes nestas condições.* (T14)

Os participantes também deram ênfase às normas excessivas, aos protocolos e rotinas diárias que devem ser cumpridas na Unidade e que acabam por mecanizar e despersonalizar o processo de trabalho e de cuidado dos profissionais de enfermagem. A rotina impensada e automatizada reduz o cuidado a um mero fazer técnico, pontual e rotineiro, desacompanhado de reflexão crítico-reflexiva e sem levar em consideração o cuidado singular e individualizado das gestantes, conforme expresso a seguir:

*Aqui na unidade desempenho um cuidado nada específico devido as rotinas estabelecidas neste setor. O tratamento não é individualizado. Tudo parece ser mecânico, técnico.* (E4)

Denotou-se em outros depoimentos, que a equipe de enfermagem não está devidamente qualificada para cuidar de gestantes internadas em uma unidade de internação hospitalar psiquiátrica, embora reconheçam que a gestante passa por diversas alterações fisiológicas e psicológicas e que esta precisa ser acolhida e amparada, não como ‘mais uma paciente’ (número), mas como alguém que vivencia um momento ímpar de sua vida e da vida de seu filho. Logo, esse cuidado singularizado à gestante é, por vezes, banalizado em detrimento das normas rígidas, protocolos e rotinas impensadas. Por vezes, a gestante é reconhecida, mas somente quando esta apresenta alguma intercorrência e/ou necessita de algum cuidado diferenciado.

*O cuidado da gestante no serviço acaba despercebido se ela não apresenta intercorrências.* (E2)

*Acredito que, assim como eu, a equipe não se encontra capacitada suficientemente.* (E4)

*Falta de preparo para este público.* (E7)

*Dificuldade com o despreparo da equipe para atendimento específico para gestante.* (E1)

*Percebo a falta de treinamento.* (A1)

Percebeu-se, ainda, nos depoimentos de alguns participantes as dificuldades relacionadas ao registro correto e completo das informações nos prontuários das gestantes em relação às especificidades do binômio mãe-feto. Esse processo, por vezes, é reduzido a uma ação secundária, dissociada do cuidado integrador e complementar, conforme expresso:

[...] *é preciso melhorar os registros. Ter mais detalhes sobre a mãe e o bebê.* (A2)

*O registro não é assumido como cuidado a ser realizado para a mãe e o bebê* [...]. (T7)

*No processo de enfermagem como instrumento para delinear a assistência não há alterações significativas entre a gestante e um paciente comum.* (E6)

*É necessária a inclusão de cuidados específicos para a gestação nas prescrições de enfermagem, que tragam informações sobre a gestante e o bebê dela* [...]. (E4)

A hospitalização, frequentemente, despersonaliza a pessoa e a reduz a um mero paciente, portador de uma doença e que necessita de tratamento. Logo, a mesma é apreendida e tratada apenas como “doente” e reduzida à dimensão física, sem considerar a multidimensionalidade humana no cuidado. Nessa lógica, todas as demais dimensões, igualmente relevantes, são relegadas a um segundo plano. Sob esse enfoque, um participante referiu que o cuidado deve ser ampliado e o ato de cuidar precisa contemplar, além da visão biológica, a pessoa/paciente em sua integralidade e multidimensionalidade. Como, contudo, ampliar essa percepção de cuidado em enfermagem/saúde?

### Da impessoalidade à singularidade do cuidado de enfermagem

Embora os participantes tenham referido pouco conhecimento e habilidade para lidar e cuidar das gestantes, denotou-se, por outro lado, o anseio para que a gestante seja acolhida, amparada e cuidada em sua singularidade e para além de sua doença mental, conforme depoimentos:

*Eu percebo que a gestante não pode ser tratada como mais uma paciente. Ela está em um momento especial de sua vida e da vida de seu filho.* (TE3)

*Considero um trabalho complexo, que necessita maior embasamento teórico-prático para auxiliar no manejo e cuidado à gestante, por ser um momento especial da vida dela.* (AI)

A gestação, na compreensão dos participantes, comporta uma dinâmica integradora que envolve o binômio mãe-bebê. Sob esse enfoque, o cuidado à gestante precisa, também, apreender e abarcar o seu filho, em gestação. Ferramentas tecnológicas e/ou procedimentos operacionais específicos que apoiem o cuidado em unidades psiquiátricas foram sinalizadas como estratégias para qualificar o processo de trabalho.

*Inexiste protocolo de rotinas e orientações especificas para o cuidado com a gestante. Teríamos que pensar algo nesta direção.* (T1)

*Sinto falta de um recurso específico para as gestantes. Elas precisam ser tratadas em sua individualidade. Estes recursos nos ajudariam a individualizar o processo de cuidado.* (E5)

Para além de procedimentos operacionais direcionados às gestantes, outros participantes mencionaram à necessidade de tecnologias que assegurem o cuidado multidimensional de enfermagem, desde o planejamento à sua execução. Reconhecem, nessa direção, que o cuidado de enfermagem demanda saberes específicos para a tomada de decisão, a partir do raciocínio crítico-reflexivo.

*Como melhorias deveriam existir protocolos para nortear as rotinas da gestante. Estes nos ajudariam na tomada de decisão.* (T1)

*Sugiro que seja proposto e estabelecida alguma tecnologia de apoio à implementação de um cuidado especializado para as gestantes internadas.* (E4)

*Falta mais planejamento e sistematização para a tomada de decisões.* (E7)

As dificuldades relacionadas à tomada de decisão sobre a melhor conduta a ser planejada e adotada com a gestante foram evidenciadas em diversos depoimentos. De modo geral, os profissionais de enfermagem manifestaram interesse em ampliar a sua percepção e reconheceram a necessidade de conhecimento específico para lidar com as gestantes internadas com transtornos mentais.

*Acredito que a maior dificuldade esteja relacionada as abordagens, de como acompanhar essa gestante no serviço, saber quais cuidados específicos utilizar na assistência. Precisamos qualificação.* (E2)

Percebeu-se, em diversos depoimentos, que os profissionais de enfermagem se sentem despreparados para lidar com as especificidades psiquiátricas das gestantes internadas. Esse pensar demonstra, que o Processo de Enfermagem necessita ser adaptado para as diferentes realidades e que os profissionais, igualmente, precisam ser instrumentalizados com base nas especificidades de cada contexto e realidade existencial.

*Acho que a assistência fica limitada, devido a gestante ter mais restrições e nós não sabermos lidar.* (T14)

*Falta de prática com esse tipo de público.* (T12)

Uma das tarefas fundamentais da condição humana é o ato de cuidar, que se consolida a partir das interações, associações, tecnologias e aprendizados ao longo da vida. Nessa perspectiva, o cuidado está imbricado na dinâmica existencial de todo e qualquer ser humano, o qual deve ser acolhido como singular em suas diversas etapas da vida. Assim, assegurar um cuidado de enfermagem singular às gestantes com transtornos mentais significa atribuir-lhes significado de vida, de existência, de saúde integral e contribuir para o desenvolvimento saudável de seu filho, em gestação.

## DISCUSSÃO

As unidades hospitalares psiquiátricas são reconhecidas, geralmente, pelo seu controle sobre rotinas diárias e determinações protocolares rígidas, as quais fragilizam as interações e as manifestações de individualidade. Nessas unidades os pacientes são, na maioria das vezes, tratados de forma genérica e impessoal e reduzidos, habitualmente, a pacientes mentais^(21)^. Esse pensar e agir vem sendo questionado, mais especificamente, a partir da Reforma Psiquiátrica que prevê um olhar integral e humanizado na saúde mental^(22)^.

Embora a qualidade e a eficácia do cuidado em saúde mental tenham conquistado novos patamares, nas últimas décadas, as revoluções terapêuticas na psiquiatria ainda seguem (re)produzindo estigmas. Estudo demonstra que o demérito relacionado ao paciente mental e a consequente fragmentação do cuidado nesta área, se constituem em barreira ao tratamento e à recuperação. Além disso, o estigma associado à doença - paciente mental - tem impactado no comportamento de procura e no viver saudável destes usuários^(23)^.

Os transtornos mentais em gestantes seguem sendo temática complexa. Além do estigma doença -paciente mental, a gestante carrega o demérito da falta de acesso específico aos serviços de saúde mental perinatais, à frágil qualificação dos profissionais da saúde nesta área específica, à falta de abordagens integrativas e colaborativas baseados em evidências científicas, dentre outros fatores. Estudo evidencia, que o estigma do transtorno mental é, sobremaneira, uma barreira à procura de apoio nos serviços de saúde e, consequentemente, a temática permanece inexplorada em grande parte dos países de baixo e médio desenvolvimento^(24)^.

Denotou-se, em outro estudo, que gestantes com doenças mentais pré-existentes apresentam risco aumentado nos resultados obstétricos e neonatais em comparação às gestantes sem transtornos mentais prévios^(25)^. A doença mental prévia em gestantes potencializa os riscos obstétricos e, portanto, demanda uma assistência singular e integral, tendo em vista que a má qualidade do cuidado pode influenciar no vínculo materno-infantil e incorrer em implicações a médio e longo prazo tanto na mãe quanto no filho, parceiros/famílias^(26)^.

Evidenciou-se, nessa mesma direção, que gestantes internadas em unidades psiquiátricas são consideradas usuárias com um maior número de doenças agudas se comparadas àquelas que são tratadas e acompanhadas em ambulatórios^(27,28)^. Inexistem investigações, contudo, que demonstram como a gravidade dos transtornos mentais em gestantes, de acordo com a qualidade da assistência recebida, pode estar associada aos resultados obstétricos adversos.

Tendo em vista a prevalência e o impacto dos transtornos mentais no período gestacional e, paralelamente, um processo de cuidado pouco singular e integralizado, tornam-se fundamentais novos estudos, de modo a clarificar os reais impactos e promover avanços relacionados às abordagens de intervenção em saúde mental. Os resultados da presente pesquisa demonstraram que existe um despreparo profissional em relação ao cuidado às gestantes internadas em unidades psiquiátricas, embora se vislumbre um desejo de qualificação na área, no sentido de contemplar e assegurar à individualidade de cada usuária(gestante).

Para além do local de cuidado à gestante com transtornos mentais, os resultados do presente estudo intuíram pontos de discussão relacionados às abordagens de intervenção por parte dos profissionais de saúde e que dizem respeito à compreensão de cuidado. O cuidado em saúde é, por excelência, um fenômeno complexo pelo seu caráter singular, original e transformador. Dinamizado por meio de múltiplas relações, interações e associações sistêmicas, o cuidado tem sempre por finalidade última o bem-estar da pessoa/usuário em âmbito individual e coletivo, independente do seu estado emocional ou dimensão acometida^(29)^.

Nessa direção ampliada, a singularidade do cuidado em enfermagem tem uma relação direta com o significado de trabalho, de ambiência, de acolhimento, de empatia e respeito às diferenças, independente da natureza da doença ou do estado emocional do paciente/usuário. O enfermeiro possui função relevante e reconhecida ao liderar, prospectivamente, o processo de cuidado, a partir de tecnologias horizontalizadas, dialógicas e colegiadas^(29)^. É necessário, para tanto, ampliar a percepção e o conhecimento sobre o real significado do cuidado em saúde, o qual deve transcender a lógica fragmentária e técnica - doença mental e alcançar a integralidade e a multidimensionalidade humana.

O cuidado de enfermagem consiste, nessa dinâmica evolutiva, em envidar esforços transpessoais e multiprofissionais em direção ao outro, nesse caso à gestante com transtornos mentais, no intuito de protegê-la, promovê-la e acolhê-la em sua singularidade, independente do seu estado, situação ou comprometimento^(30,31)^. Esse pensar implica em ir além da percepção biológica de assistência em saúde e, prospectivamente, alcançar um pensamento integrador no qual o cuidador/profissional perceba-se no cuidado e como cuidado.

Estudo^(32)^ demostra, que apesar de ser um conceito complexo, o cuidado ampliado e multidimensional apreende a pessoa humana em sua singularidade. Sob esse enfoque, o cuidado fornece uma compreensão profunda de cada paciente/usuário, a partir de suas necessidades e demandas específicas. Nessa direção, além de contribuir para a satisfação dos usuários, o mesmo resulta em bem-estar individual e coletivo.

A qualidade e o impacto do cuidado de enfermagem são determinados, em suma, pela qualidade das relações e interações e o respeito às singularidades humanas. Estudo^(29)^ reforça esse pensar, ao considerar que o cuidado é determinado pelo acolhimento respeitoso e singular às necessidades de cada usuário, família, comunidade, independente da situação em que se encontram.

### Limitações do estudo

As limitações deste estudo estão associadas à não participação de todos os profissionais da equipe de enfermagem convidados para este fim, considerando que os resultados poderiam ter sido bem mais desenvolvidos e sólidos. A sua não participação pode estar relacionada à sobrecarga de atividades em decorrência do período pandêmico. Outra limitação pode estar associada ao fato deste estudo ter sido realizado em uma única unidade psiquiátrica, o que inviabiliza generalizações.

### Contribuições para a área da Enfermagem

O estudo contribuirá para a organização da assistência de enfermagem às gestantes internadas em unidades hospitalares psiquiátricas, a partir de abordagens singulares e multidimensionais. Desse modo, os resultados deste estudo poderão alavancar novas estratégias de gestão, em âmbito do Conselho Federal de Enfermagem (COFEN), para intuir o Processo de Enfermagem nestas unidades específicas, frequentemente, desprovidas de estudos, qualificações e tecnologias específicas.

## CONSIDERAÇÕES FINAIS

A organização da assistência de enfermagem às gestantes internadas em unidade hospitalar psiquiátrica, na percepção da equipe de enfermagem, se traduz em movimento que envolve técnicas, rotinas e protocolos. O cuidado de enfermagem às gestantes, em situação de internação psiquiátrica, demanda contínua qualificação profissional, tecnologias interativas e de apoio ao processo de enfermagem, além da promoção de um cuidado singular e multidimensional.

Assegurar um cuidado de enfermagem singular às gestantes, sobretudo, às internadas em unidades psiquiátricas, significa atribuir-lhes significado de vida, de existência e conduzir o cuidado, a partir de uma perspectiva multidimensional e multidisciplinar, a fim de assegurar a integralidade e a continuidade do cuidado em saúde. Significa repensar abordagens e conduzir o cuidado, a partir dessas perspectivas. Consiste, em suma, pensar o cuidado de forma colaborativa e interconectada com os demais níveis de complexidade, a fim de assegurar a integralidade e a continuidade do cuidado em saúde.
